# Significance Support Vector Regression for Image Denoising

**DOI:** 10.3390/e23091233

**Published:** 2021-09-20

**Authors:** Bing Sun, Xiaofeng Liu

**Affiliations:** 1 State Key Laboratory of Mechanical Transmissions, Chongqing 400044, China; sunbing@cqu.edu.cn; 2 College of Mechanical Engineering, Chongqing University, Chongqing 400044, China

**Keywords:** support vector regression, sample significance, sample density, cutoff distance, image denoising

## Abstract

As an extension of the support vector machine, support vector regression (SVR) plays a significant role in image denoising. However, due to ignoring the spatial distribution information of noisy pixels, the conventional SVR denoising model faces the bottleneck of overfitting in the case of serious noise interference, which leads to a degradation of the denoising effect. For this problem, this paper proposes a significance measurement framework for evaluating the sample significance with sample spatial density information. Based on the analysis of the penalty factor in SVR, significance SVR (SSVR) is presented by assigning the sample significance factor to each sample. The refined penalty factor enables SSVR to be less susceptible to outliers in the solution process. This overcomes the drawback that the SVR imposes the same penalty factor for all samples, which leads to the objective function paying too much attention to outliers, resulting in poorer regression results. As an example of the proposed framework applied in image denoising, a cutoff distance-based significance factor is instantiated to estimate the samples’ importance in SSVR. Experiments conducted on three image datasets showed that SSVR demonstrates excellent performance compared to the best-in-class image denoising techniques in terms of a commonly used denoising evaluation index and observed visual.

## 1. Introduction

A broad variety of digital images are widely used in applications such as medicine, remote sensing, military, and robotics [1]. Owing to the limitations of the acquisition equipment and the acquisition environment, images are inevitably affected by noise during the acquisition process. The reduction of image noise is an essential basis for image processing and helps in enhancing the performance of image statistics and processing. In recent years, several denoising approaches have been proposed to mitigate the ill effects of signal distortion by noise and strengthen procedures for image inspection and information extraction [2]. As a typical linear non-local filter, Gaussian filtering (GF) uses the correlations among the entire pixel range of an image [3]. GF has a capacity for edge preservation, but the filter weights become lighter as the distance from the center increases, leading to the blurring of edges. To suppress blurring and localization problems caused by the linear diffusion filter, Perona and Malik proposed an anisotropic diffusion filter (ADF) using partial differential equations [4]. ADF utilizes a non-linear diffusion that avoids the loss of edges but is sensitive to noise, over-blurring, and boundary sharpening, which may however cause the loss of details. Furthermore, wavelet-based denoising methods with properties of sparsity and multiresolution are widely used in signal denoising [3]; however, these methods cannot handle image anisotropic features such as edges owing to poor effectiveness in optimally representing multivariate functions [5]. The drawbacks of wavelet transformation (WT) denoising methods are demonstrated by ringing artifacts caused by WT tending to add extra edges or structures into the image. There are also several modeling methods that have been applied in image denoising by investigating model parameters from prior information with a noisy image, such as local complexity estimation based filtering [6], Bayesian inference [7], sparse representation [8], hidden Markov models [9], and low rank matrix restoration [10]. However, these methods have the same drawbacks of over-smoothing or edge sharpening. Recently, deep learning-based methods have been widely researched in the field of image denoising, but most of these methods suffer from performance saturation or high computational cost [11]. Soft computing techniques based on fuzzy logic are also often adopted for researching the uncertain influences of images [12,13].

As a generalization of SVM, SVR attempts to find the flattest region containing most of the training instances in the feature space. By constructing an optimal objective with the loss function and the geometrical properties of the region, SVR predicts actual values according to the structural risk minimization principle [14]. During the past decade, SVR has been widely expanded for application in various research fields, including energy conservation [15], time series forecasting [16], and system evaluation on remaining useful life [17]. With regard to image processing, Zhi et al. offered a denoising method with SVR by improving the kernel function with a three-domain fuzzy function [18]. Li et al. applied SVR with blind image deconvolution for image denoising, and their experiments demonstrated the robustness of SVR for unknown level noise [19]. Cheng et al. introduced an image denoising method based on wavelets and SVR and achieved good performance on real images contaminated by Gaussian noise [20]. However, all of the above-mentioned SVR improvements consider all samples to be equally important. We note that ignoring the changing of samples distribution caused by noise generally leads to regression deviation.

To increase noise robustness, some sample prior information is used to improve the performance of SVR. Qiang et al. introduced a robust weighted linear loss SVR for large-scale classification by punishing the rest class samples [21]. Luo et al. improved double-weighted SVR combined with transfer learning for small datasets, which reduced the effect of sample bias and improved prediction performance [22]. Wu et al. provided weighted multiscale SVR for ultraviolet-visible spectroscopy, in which the weights were calculated by empirical mode decomposition and used for sub-SVR processing [23]. Gu et al. proposed fast clustering-based weighted twin SVR, where a fast clustering algorithm was adopted to obtain a weighted diagonal matrix for several categories [24]. However, some studies only consider the distribution information among different clustering. In addition, some of the above-mentioned distribution information was obtained with a kernel-based distance influenced by the choice of multiple kernel parameters. On the other hand, due to its formal limitations, it is only applicable on limited datasets and loses the flexibility and universality of the model.

It is noteworthy that the noise information of pixels is critical in image denoising. Considering the high relevancy of neighborhood pixels and the disturbance of noise, obtaining the samples significance estimation remains challenging. In this paper, the SSVR method is proposed with optimized noise robustness and applied in image denoising. The significance factor based on spatial density information is calculated for each sample pixel to measure the noise information of images. Then, the significance factor is adopted as a weight coefficient of the penalty parameter in SVR for reducing noise interference in the model training process. The contributions of this paper are summarized as follows.

(1) A significance estimation framework based on spatial density information is introduced to evaluate sample importance.
(2) SSVR is proposed as an improved SVR with sample significance. In SSVR the significance factors are attached to samples as the weight coefficients during model training.
(3) As an implementation of SSVR with sample spatial density, cutoff distance-based SSVR with enhanced noise robustness is successfully applied in image denoising with remarkable results.

## 2. Methods

### 2.1. SVR

Given a training dataset {*x_i_*, *y_i_*} (*i* = 1, 2, …, *l*), where *x_i_* is a sample data vector, and *y_i_* is its corresponding value. Then, the goal of SVR is to minimize the sum of the squared errors [25].

(1) $$\begin{array}{l} \underset{w,b}{\min}\frac{1}{2}\sum_{i=1}^{l}{\left\|w\right\|}^{2}\\ s.t.\begin{array}{c} w^{T}\phi (x_{i})+b-y_{i}\leq \varepsilon \\ y_{i}-w^{T}\phi (x_{i})-b\leq \varepsilon \end{array} \end{array}$$

From a geometric point of view, the learning goal of SVR is to find a tunnel in an *N*-dimensional data space. The position of the tunnel is determined by a hyperplane with normal vector *w* and bias vector *b.* The width of the tunnel is denoted by *ε*. Kernel *ϕ*(*x_i_*) is introduced to enhance the non-linear regression capability of SVR.

The objective function (1) is called the hard margin SVR. Obviously, the objective function (1) has poor generalization performance because it is not possible to have no outliers without affecting the results. To limit overfitting and to increase the applicability of the objective function on new data, a soft margin SVR is proposed by introducing a regularization term into the objective function.

(2) $$\begin{array}{l} \min\frac{1}{2}w^{T}w+C\sum_{i=1}^{l}\left(\xi_{i}+\xi_{i}^{*}\right)\\ s.t.\begin{array}{c} w^{T}\phi (x_{i})+b-z_{i}\leq \varepsilon +\xi_{i}\\ z_{i}-w^{T}\phi (x_{i})-b\leq \varepsilon +\xi_{i}^{*}\\ \xi_{i},\xi_{i}^{*}\geq 0,\;i=1,\ldots ,l \end{array} \end{array}$$

The relaxing $\xi_{i},\xi_{i}^{*}\geq 0,i=1,2,\cdots ,l$ is introduced to improve the generalization capability, and *C* > 0 is the penalty factor. Here, $\xi_{i}$ and $\xi_{i}^{*}$ are the lower training errors.

### 2.2. Sample Significance Estimation

Considering the space distribution of training samples, it is obvious that normal samples should have better aggregation, i.e., a larger sample density, while outliers caused by noise generally deviate from the overall sample distribution and therefore have a smaller sample density. Based on this idea, in order to better measure the significance of samples in the model training process, this paper proposes sample significance established by utilizing sample spatial density. Adapting sample spatial aggregation to define sample spatial density is an intuitive method. For dataset $\left\{x_{i}\in X,i=1,2,\cdots N\right\}$, the neighborhood parameter is *ε*, and the sample distance measure is *dis*(*x_i_*, *x_j_*), then its ε-neighborhood sample set is defined as:

(3) $$N_{\varepsilon }(x_{i})=\left\{x_{i}\in X|dis(x_{i},x_{j})<\varepsilon \right\}$$

and the number of samples in the sample set is defined as:

(4) $$\left|N_{\varepsilon }(x_{i})\right|=\sum_{j}\chi (dis(x_{i},x_{j}-\varepsilon ))$$

where χ(t)=1 if *t* < 0 and otherwise χ(t)=0.

Then, from the view of measuring the relative importance of samples by utilizing sample spatial aggregation, the significance is defined as:

(5) $$S(x_{i})=\left|N_{\varepsilon }(x_{i})\right|/\max(\left|N_{\varepsilon }(x_{i})\right|)$$

**Figure 1:**
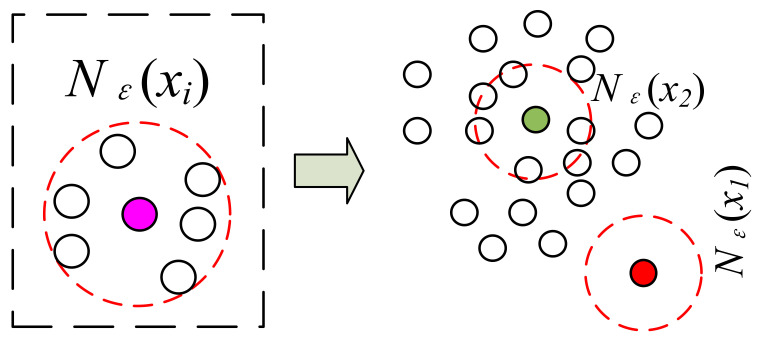
Sample significance based on spatial aggregation.

As shown in Figure 1, the left part shows the sample neighborhood *N_ε_*(*x_i_*). The red dashed circle is the *ε*-neighborhood sample set *N_ε_*(*x_i_*) of the pink sample and |*N_ε_*(*x_i_*)| is 6. As can be seen from the right part of Figure 1, the red point has a small number of samples in its sample set *N_ε_*(*x_i_*) due to its deviation from the sample population, while the green point has high sample aggregation, and therefore has a large number of samples in its sample set *N_ε_*(*x_i_*) and a large value of |*N_ε_*(*x_i_*)|.

From the definition of sample significance, it is clear that the sample significance takes a range of values (0, 1]. The more aggregated the sample, the larger the value of significance *S*(*x_i_*). In addition, the more deviated the sample from the group, the smaller the value of significance *S*(*x_i_*). This property can also be visualized in Figure 1.

In practical application, different types of distances in high-dimensional space can be chosen so that the sample significance in high-dimensional space can be estimated. Obviously, the proposed sample significance is related to the choice of appropriate definition of spatial aggregation degree information. Sample significance obtained from different distribution models or sample aggregation methods is different. These precisely reflect the flexibility of the proposed sample significance model, which can be applied to different application areas according to different prior knowledge.

### 2.3. SVR with Sample Significance (SSVR)

From the solution process of SVR, it can be seen that SVR gives the same attention to all samples, i.e., the same penalty factor C is given to each sample in the objective function of SVR. In fact, outliers caused by noise should be given lower attention when training the model to reduce the impact of outliers on the regression model.

By introducing significance estimation *S*(*x_i_*) as a weight coefficient, the objective function and constraint condition of SSVR are as follows:

(6) $$\begin{array}{l} \min\frac{1}{2}w^{T}w+S(x_{i})C\sum_{i=1}^{l}\left(\xi_{i}+\xi_{i}^{*}\right)\\ s.t.\begin{array}{c} w^{T}\phi (x_{i})+b-y_{i}\leq \varepsilon +S(x_{i})\xi_{i}\\ y_{i}-w^{T}\phi (x_{i})-b\leq \varepsilon +S(x_{i})\xi_{i}^{*}\\ \xi_{i},\xi_{i}^{*}\geq 0,\;i=1,\ldots ,l \end{array} \end{array}$$

Similar to the standard SVR, the Lagrange function is introduced with Lagrange multipliers $\alpha_{i}\geq 0,\alpha_{i}^{*}\geq 0$ and $\beta_{i}\geq 0,\beta_{i}^{*}\geq 0$. Then, the dual problem of Equation (6) can be expressed as follows:

(7) $$\begin{array}{l} L(w,b,\xi ,\xi^{*},\alpha ,\alpha^{*},\beta ,\beta^{*})\\ =\frac{1}{2}{\left\|w\right\|}^{2}+S(x_{i})C\sum_{i=1}^{l}\left(\xi_{i}+\xi_{i}^{*}\right)\\ -\sum_{i=1}^{l}\beta_{i}S(x_{i})\xi_{i}-\sum_{i=1}^{l}\beta_{i}^{*}S(x_{i})\xi_{i}^{*}\\ +\sum_{i=1}^{l}\alpha_{i}(w^{T}\phi (x_{i})+b-z_{i}-\varepsilon -S(x_{i})\xi_{i})\\ +\sum_{i=1}^{l}\alpha_{i}^{*}(z_{i}-w^{T}\phi (x_{i})-b-\varepsilon -S(x_{i})\xi_{i}^{*}) \end{array}$$

Setting the partial derivatives of *L* with respect to *w*, *b*, *ξ*, and ξ^*^, respectively, and ensuring that Karush–Kuhn–Tucker (KKT) conditions are met in the above process, it can be obtained that:

(8) $$\begin{array}{l} w=\sum_{i=1}^{l}(\alpha_{i}^{*}-\alpha_{i})x_{i},0=\sum_{i=1}^{l}(\alpha_{i}^{*}-\alpha_{i}),\\ S(x_{i})C=\alpha_{i}+\beta_{i},S(x_{i})C=\alpha_{i}^{*}+\beta_{i}^{*} \end{array}$$

By replacing constraints (8) with (7) and rearranging the optimization function, we can obtain the dual problem solution.

(9) $$\begin{array}{l} \max(\sum_{i=1}^{l}y_{i}(\alpha_{i}^{*}-\alpha_{i})-\varepsilon (\alpha_{i}^{*}-\alpha_{i})\\ -\frac{1}{2}\sum_{i=1}^{l}\sum_{j=1}^{l}\left(\alpha_{i}^{*}-\alpha_{i})(\alpha_{j}^{*}-\alpha_{j}\right)\phi {\left(x_{i}\right)}^{T}\phi (x_{i}))\\ s.t.\begin{array}{c} \sum_{i=1}^{l}\left(\alpha_{i}^{*}-\alpha_{i}\right)=0\\ 0\leq \alpha_{i}^{*},\alpha_{i}\leq S(x_{i})C \end{array} \end{array}$$

Then. bringing the first term of Equation (8) into the decision function in Equation (6) yields a final decision function:

(10) $$\begin{array}{l} f(x)=\sum_{i=1}^{l}({\overset{⌢}{\alpha }}_{i}-\alpha_{i}\langle \phi (x_{i}),\phi (x_{j})\rangle -b\\ b=z_{i}+\varepsilon -\sum_{i=1}^{l}({\overset{⌢}{\alpha }}_{i}-\alpha_{i})\langle \phi (x_{i}),\phi (x_{j})\rangle \end{array}$$

From the above derivation process, it can be seen that the solutions of SSVR are almost the same as SVR.

Traditional SVR intends to find a minimum tunnel in a feature space that contains all the sample points. *ε* is a threshold specifying the width of the tunnel. In Equation (1), because there is no effect on regression interval, any training data that lie within the *ε*-intensive area are ignored by the objective function. In the optimization objective, the first term is used to describe the size of the interval between the regression hyperplanes, i.e., the structural risk, and the second term describes the error on the training set, i.e., the empirical risk. *C* denotes a compromise between the two items. A larger *C* indicates that the optimization objective places more emphasis on the risk of missing samples and tries not to miss any samples in the regression, which may lead to a very large regression area and overfitting. In contrast, a smaller *C* indicates that more emphasis is placed on the highest priority of the interval, and some training samples may be missed in the regression as appropriate. By introducing relaxing factors, the soft margin SVR increases the tolerance of mislabeled training samples. In other words, within the relaxing factor *C*, the regularization term constrains the area to be as flat as possible.

The differences between SVR and SSVR are the upper ranges of $\alpha_{i}^{*}$ and $\alpha_{i}$. Unlike standard SVR, in which the penalty term *C* is a fixed value, SSVR applies the sample significance coefficient *S*(*x_i_*) to the penalty term *C*. Thus, in SSVR, the upper limit of $\alpha_{i}^{*}$ and $\alpha_{i}$ is a refined boundary. In practical applications of SVR, the training data are often subject to noise or outliers that often become support vectors in the SVR training process. In such cases, the regression planes and bounds can deviate significantly from the optimal solution. This phenomenon is known as the outlier sensitivity problem of the standard SVR algorithm [26]. As shown in Equation (2) of the standard SVR, the penalty term *C* determines the importance of the SVR objective function in treating outliers. Traditional SVR treats all training data points equally during training. Because SVR minimizes the distance to the farthest sample point from the regression hyperplane, outliers with long distances can lead to large regression errors in SVR due to an excessive trade-off. The SSVR weighted by refined *C* reduces the influence of less important data points (e.g., outliers or noise). This reduces the influence of the trade-off part caused by outliers in the objective function of the SVR.

### 2.4. Influence of Significance Factor for SSVR

Given a dataset, {*x_i_*, *i* = 1, 2, …, *l*} is the input vector and *y_i_* is the output in the SVR model*_._* Particularly in image processing using SVR, the input *x_i_* denotes the vector constructed with a center pixel *i* and its neighborhood. The output *y_i_* denotes the value of pixel *i* of the original image [27]. Then, the experimental risk is:

(11) $$\begin{array}{l} R_{emp}[f]=\frac{1}{N}\sum_{i=1}^{N}(\xi_{i}+\xi_{i}^{*})f(x_{i},y_{i})\partial_{x\times y}\\ =\frac{1}{N}\partial_{x\times y}\sum_{i=1}^{N}f(x_{i},y_{i})(\xi_{i}+\xi_{i}^{*}) \end{array}$$

where $\partial_{x,i}$ is the unit hypercube sample distribution in the data space *f*(*x_i_*, *y_i_*), expressing the density distribution of (*x_i_*,*y_i_*).

From the experiential risk minimization defined in Equation (11), obviously, the sparser the data distribution, the lower *f*(*x_i_*, *y_i_*) will be. The sparsity of data distribution also influences the training error $\xi_{i}+\xi_{i}^{*}$, which is weighted by *f*(*x_i_*, *y_i_*). Hence, the sample significance can be adopted to measure the density information of (*x_i_*,*y_i_*) to minimize experiential risk during the training process.

In SSVR, the significance factor is *S*(*x*). Hence, the experimental risk is:

(12) $$\begin{array}{l} R_{emp}[f]=\frac{1}{N}\sum_{i=1}^{N}(\xi_{i}+\xi_{i}^{*})S(x_{i})\partial_{x\times y}\\ =\frac{1}{N}\partial_{x\times y}\sum_{i=1}^{N}S(x_{i})(\xi_{i}+\xi_{i}^{*}) \end{array}$$

Let *h* be the Vapnik–Chervonenkis dimension of candidate sets of decision functions *F* and δ∈(0,1]. Then, if

(13) $$\begin{array}{l} N>h,\\ h(\ln\frac{2N}{h}+1)+\ln\frac{4}{\delta }\geq \frac{1}{4} \end{array}$$

For significance weight *S*(*x*) and δ∈(0,1], there exists an inequality holding with a probability no less than 1−δ.

(14) $$R[f]\leq R_{emp}[f]+\sqrt{\frac{8}{N}\left[h(\ln\frac{2N}{h}+1)+\ln\frac{4}{\delta }\right]}$$

where *R*[*f*] is the structure risk, and *R_emp_*[*f*] represents the empirical risk. Substituting Equation (13) into Equation (14), we obtain

(15) $$\begin{array}{l} R[f]\leq (\frac{1}{N}\partial_{U\times y}S(x_{i})\sum_{i=1}^{N}\left(\xi_{i}+\xi_{i}^{*}\right)\\ +\sqrt{\frac{8}{N}\left[h(\ln\frac{2N}{h}+1)+\ln\frac{4}{\delta }\right]}) \end{array}$$

In this study, the empirical risk is minimized using SSVR. If the number of samples is sufficiently large, it is reasonable to assume that $\lim_{N\to \infty }R_{emp}[f]=R[f]$ will hold.

### 2.5. SSVR with Cutoff Distance-Based Sample Significance and Noise Robustness Example

As an example, this paper implements a cutoff distance-based SSVR and applies it to image denoising. In application, the more accurate the priori information is, the more effective the improvement of the noise robustness of the SSVR model. From the intuitive two-dimensional data example, the sample significance introduction can better improve the noise denoising effect than traditional SVR.

Considering a dataset $x_{i}\in X,i=1,2,\cdots ,N$, the Euclidean distance between *x_i_* and *x_j_* is stored in vector *D_sort_* in order from small to large. The cutoff distance *ε* is defined following [28]:

(16) $$\varepsilon_{cutoff}=D_{sort}(0.2*N)$$

The *ε*-neighborhood sample set *N_ε_*(*i*) is defined as:

(17) $$N_{cutoff}(i)=\left\{x_{j}|dis(x_{i},x_{j})<D_{sort}(0.2*N)\right\}$$

Then the local spatial density |*N_ε_*(*i*)| of the data point *i* is:

(18) $$\left|N_{cutoff}(i)\right|=\sum_{j}\chi (d_{ij}-D_{sort}(0.2*N))$$

Hence, |*N_cutoff_*(*i*)| is the number of points that have a distance to point *i* less than the value of *D_sort_*(0.2*N*). Because the method is only sensitive to the relative value of the samples’ distance, the choice of *ε_cuotff_* is robust.

Considering that the spatial density |*N_cutoff_*(*i*)| could describe the distribution of points in the sample space, the cutoff distance-based local density significance factor can be estimated as follows:

(19) $$S(x_{i})=\left|N_{cutoff}(x_{i})\right|/\max(\left|N_{cutoff}(X)\right|)$$

The significance value is in the range of (0,1]. According to the definition, points with better aggregation have high significance, while outliers have low significance.

To visually describe the influence of noise data and the corrective capacity of the cut-off distance-based sample significance for standard SVR, two-dimensional data are regressed using SVR with a linear kernel. The values of *C* and ε here are set to 1 and 0.15, respectively.

**Figure 2:**
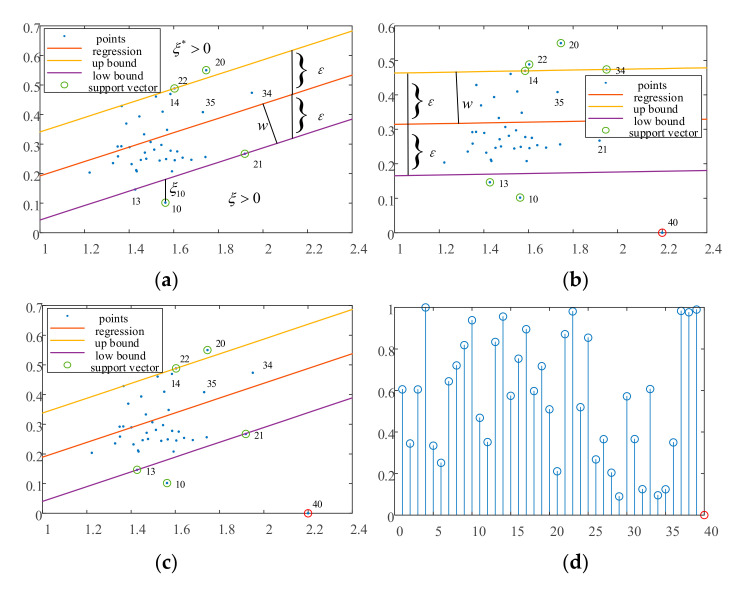
(**a**) Regression of SVR with normal data. (**b**) Regression of SVR with noised data. (**c**) Regression of SSVR with noised data. (**d**) Weights of each data sample in SSVR.

Figure 2a shows the regression for the 39 normal sample points with SVR in which the upper and lower boundaries roughly contain all the sample points, and the points 10, 20, 21, and 22 near the boundaries are support vectors. The upper boundary passes through point 22, and the lower boundary passes through points 13 and 21. Figure 2b shows the regression result of the SVR after adding an outlier point 40 at the coordinates (2.2, 0). It can be seen that the SVR regression deteriorates due to the influence of the outlier point. As the outlier point 40 becomes a support vector, it leads to an overall downward shift of the regression boundaries and normal point 20 near the upper boundary in Figure 2a, further away from the upper boundary. In contrast, because the function distance of the regression plane to the boundary is *ε*, its geometric distance is εw, where *w* is the normal vector of the hyperplane. Because both the function distances of SVR and SSVR are *ε* in the vertical direction in Figure 2a,b, the slopes of the regression planes differ, resulting in a larger geometric distance εw in Figure 2b than that in Figure 2a. This means that the outlier causes the regression interval to become larger and less compact. Figure 2c displays the results of the SSVR regression using the introduced density information. The regression intervals and location for the two methods in Figure 2a,c are almost identical. Similar to Figure 2a, the upper boundary line crosses point 22, and the lower boundary crosses points 13 and 21. Although point 40 is a support vector, its penalty parameter is very small in the objective function owing to the weighting factor. Therefore, outlier point 40 does not affect the final solution of the SVR. It can be concluded that the introduction of significance information reduces the empirical risk of the objective function and allows the objective function to focus on the structural risk. In other words, the objective function solution results in a small and compact regression interval. Figure 2d shows the significance values for all sample points. We can see that outlier 40 has the lowest significance, that is, it has the least influence on the optimization objective.

## 3. Experimental Results

In this section, gray and color images are applied to test the denoising performance of our proposed method. The experiments are carried out on the USC-SIPI dataset and then on images of real scenes. All pixel values of the images are normalized to [0, 1] in all experiments. To compare performance on image denoising, the peak signal-to-noise ratio (PSNR) and signal-to-noise ratio (SNR) are used as follows.

(20) $$PSNR=10\log_{10}\frac{\sum_{i=1}^{N}255^{2}}{\sum_{i=1}^{N}{\left(\vartheta (i)-\overset{⌢}{\vartheta }(i)\right)}^{2}}$$

(21) $$SNR=10\log_{10}\frac{\sum_{i=1}^{N}{\left(\vartheta (i)\right)}^{2}}{\sum_{i=1}^{N}{\left(\vartheta (i)-\overset{⌢}{\vartheta }(i)\right)}^{2}}$$

where ϑ(i,j) is the value of (*i*, *j*) pixel in the original image and $\overset{⌢}{\vartheta }(i,j)$ is the corresponding predicted value in the denoised image.

### 3.1. Denoising USC-SIPI Images

The images CAMERAMAN, LENA, and PEPPER from USC-SIPI are used in the experiments because they are commonly used as image processing benchmarks. CAMERAMAN is used to train the models for all methods to denoise LENA and PEPPER. The value of each pixel of the images is adopted as a feature, and the size of the sample windows is 3×3. After vectorization, the input of SVM and SSVR is a 9×1 vector.

In order to establish a comparative study, SVR from Libsvm [29] and a Gaussian filter are used as baselines. The 5-fold cross-validation method is used to search for the optimal parameters of all methods to obtain an unbiased statistical result. The RBF kernel is applied in SVR and SSVR with cutoff distance by choosing both the width parameter *σ* and trade-off parameter *C* in range (0, 1000], because it has been demonstrated that the RBF kernel usually outperforms other kernels [30]. The loss function parameter *ε* is in the range (0, 1].

**Figure 3:**
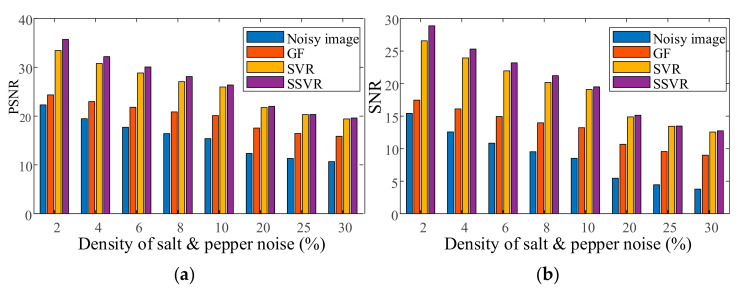
(**a**) Average PSNR of different methods. (**b**) Average SNR of different methods.

CAMERAMAN has mixed salt and pepper noise with a density of 4% as a training image. LENA and PEPPER images have mixed noise with a density from 2% to 30%. The training and testing images are denoised by GF, wavelet-based method, SVR, and the proposed SSVR method. The PSNR/SNR of the denoising results on the two images are averaged and shown in Figure 3. It is notable that CAMERAMAN, PEPPER, and LENA are all quite different from one another.

As shown in Figure 3, the proposed SSVR is superior to GF and SVR in terms of both PSNR/SNR and different salt and pepper noise densities. In general, the GF method is the least effective, with only a small increase in PNSR/SNR. At low noise density levels (2% to 6%), SSVR’s denoising performance is much better than that of SVR. As the noise density increases, the difference between SSVR and SVR’s denoising performance decreases. This is because, as the noise density increases, the noise tends to be uniformly distributed, and the weight difference between the noisy and normal samples decreases. However, even under high noise conditions, SSVR still performs better than SVR.

**Figure 4:**
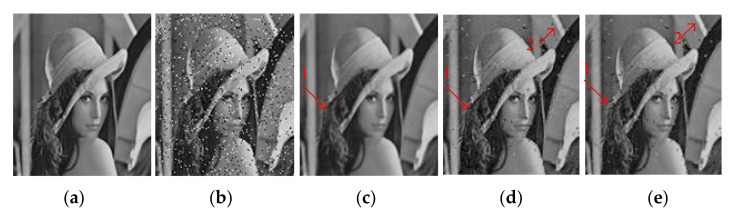
Comparison of LENA image. (**a**) Original image. (**b**) Noisy image with 6% salt and pepper noise density, PNSR = 17.65, SNR = 10.76. (**c**) Denoised image by GF, PSNR = 26.47, SNR = 19.58. (**d**) Denoised image by SVR, PSNR = 28.73, SNR = 21.85. (**e**) Denoised image by SSVR, PSNR = 29.98, SNR = 23.11.

**Figure 5:**
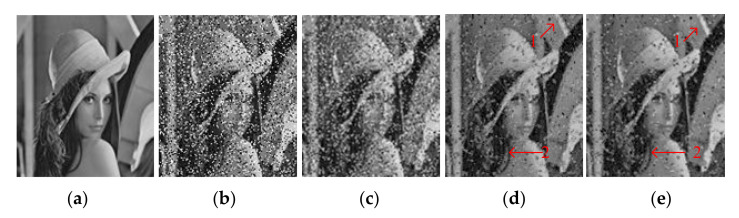
Comparison of LENA image. (**a**) Original image. (**b**) Noisy image with density 20% salt and pepper noise, PNSR = 12.45, SNR = 5.57. (**c**) Denoised image by GF, PSNR = 17.44, SNR = 10.55. (**d**) Denoised image by SVR, PSNR = 21.8, SNR = 14.9. (**e**) Denoised image by SSVR, PSNR = 22.7, SNR = 15.38.

Figure 4 and Figure 5 show the denoising effects of GF, SVR, and SSVR for different salt and pepper noise densities. As can be seen in Figure 4c, GF loses a lot of detail for Position 1. In Figure 4d,e, at the same Position 2, the white belt in Figure 4e exceeds those in Figure 4d in terms of clarity and color depth reproduction. For a noise density of 20%, the face is almost unrecognizable after GF denoising. In the comparison of the corresponding Positions 1 and 2 in Figure 5d,e, the color depth in Position 1 in Figure 5e is closer to that of the original, and Position 2 in Figure 5e has higher clarity.

### 3.2. Denoising Captured Images

**Figure 6:**
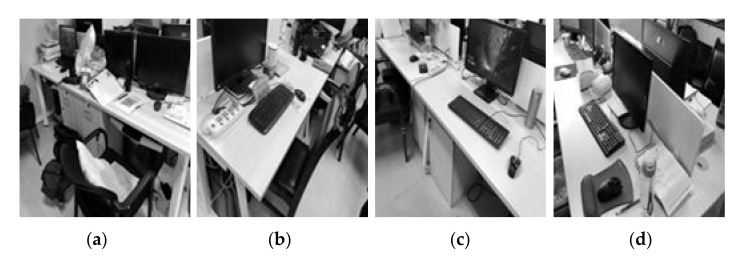
(**a**) Image1; (**b**) Image2; (**c**) Image3; (**d**) Image4.

The proposed SSVR method is used to process an image of an indoor office table, for comparison with the wavelet-based denoising method and SVR. Figure 6a shows different levels and types of noise as training images, and Figure 6b–d shows testing images.

**Figure 7:**
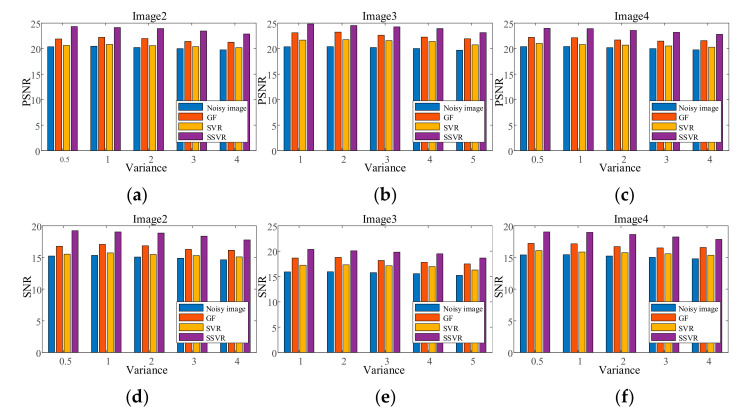
PSNR of different denoising methods with Gaussian noise under different variances on (**a**) Image2, (**b**) Image3, (**c**) Image4. SNR of different denoising methods with Gaussian noise under different variances on (**d**) Image2, (**e**) Image3, (**f**) Image4.

**Figure 8:**
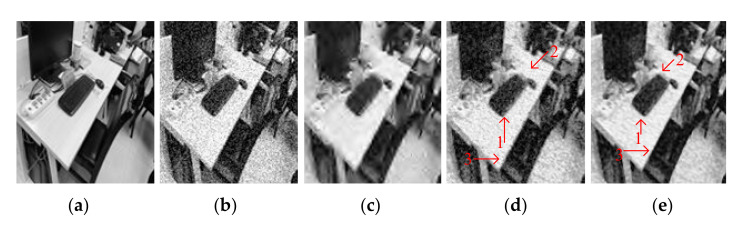
Comparison on Image2. (**a**) Original image. (**b**) Noisy image with Gaussian noise of variance 0.04, PNSR = 19.76, SNR = 14.69. (**c**) Denoised image by GF, PSNR = 22.38, SNR = 17.32. (**d**) Denoised image by SVR, PSNR = 21.13, SNR = 16.07. (**e**) Denoised image by SSVR, PSNR = 23.68, SNR = 18.62.

**Figure 9:**
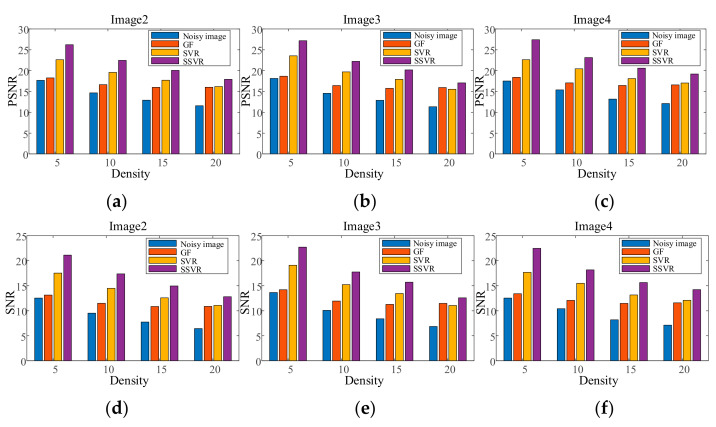
PSNR of different denoising methods with salt and pepper noise under different densities on (**a**) Image2, (**b**) Image3, (**c**) Image4. SNR of different denoising methods with salt and pepper noise under different densities on (**d**) Image2, (**e**) Image3, (**f**) Image4.

**Figure 10:**
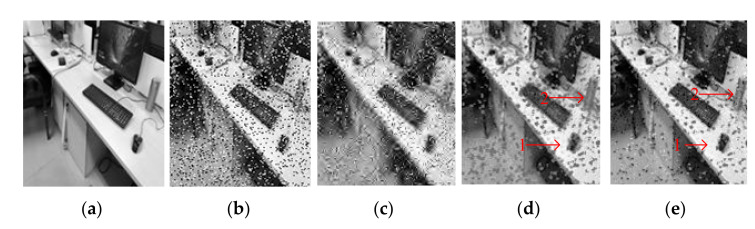
Comparison on Image3. (**a**) The original image. (**b**) Noisy image with salt and pepper with density 15%, PNSR = 12.88, SNR = 8.45. (**c**) Denoised image by GF, PSNR = 16.66, SNR = 12.23. (**d**) Denoised image by SVR, PSNR = 18.66, SNR = 14.22. (**e**) Denoised image by SSVR, PSNR = 20.39, SNR = 15.95.

**Figure 11:**
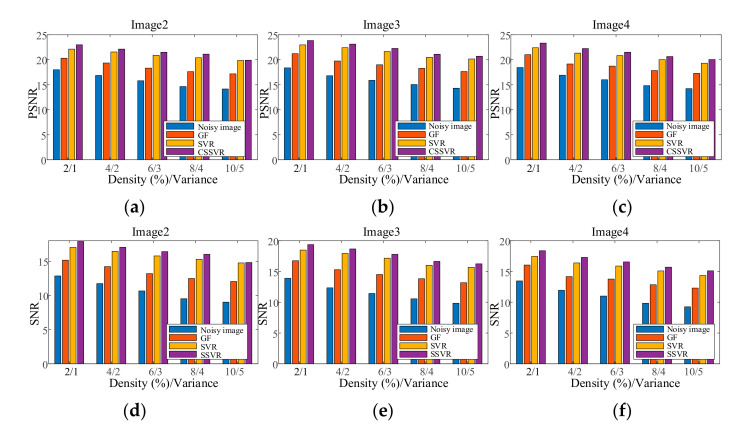
PSNR of different denoising methods mixed noise under different levels on (**a**) Image2, (**b**) Image3, (**c**) Image4. SNR of different denoising methods mixed noise under different levels on (**d**) Image2, (**e**) Image3, (**f**) Image4.

**Figure 12:**
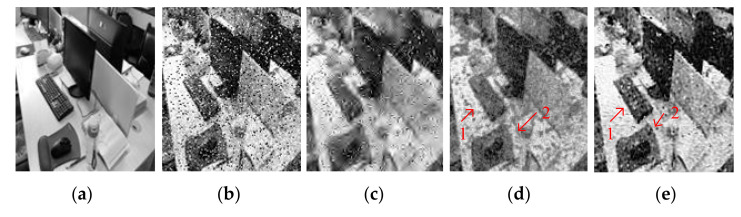
Comparison on Image4. (**a**) Original image. (**b**) Noisy image with Gaussian noise of variance 0.04 and salt and pepper with density 10%, PNSR = 13.77, SNR = 8.87. (**c**) Denoised image by GF, PSNR = 17.65, SNR = 12.76. (**d**) Denoised image by SVR, PSNR = 17.9, SNR = 13.01. (**e**) Denoised image by SSVR, PSNR = 19.05, SNR = 14.17.

Figure 7, Figure 8, Figure 9, Figure 10, Figure 11 and Figure 12 show the denoising performance and intuitively illustrations of different methods with different types and levels of noise. As can be seen, GF and SVR perform differently under different noise types. Under the Gaussian noise shown in Figure 7, GF outperforms SVR, while the opposite is true for the salt and pepper noise and mixed noise, as shown in Figure 7 and Figure 9. Furthermore, GF and SVR have different denoising effects on different images.

It can be seen that the introduction of weight distribution information leads to a greater improvement in SSVR of the denoising performance on image details.

Figure 8 shows the denoising results of the testing images with Gaussian noise added with a variance of 0.04. Figure 9c is very blurred after the GF method. By comparing Positions 1 and 2 of Figure 8d,e, it can be seen that Figure 8e is much clearer in terms of the outline of the objects. At Position 3, the two objects in Figure 8e appear slightly more three-dimensional.

Figure 10 shows test Image3 after contamination with 15% density salt and pepper noise. It can be seen from Figure 10c that the GF effect is the worst and only provides a difference in color depth. In a comparison between Figure 10d,e the outline of the object can be seen more clearly at Positions 1 and 2 in Figure 10e. Meanwhile, the top left of Figure 10e is clearer than in Figure 10d, and the color is closer to the original Figure 10a.

Figure 12 shows the testing Image4 with the addition of mixed noise, which can be seen as a double uncertainty and is difficult to handle with conventional methods. As can be seen in Figure 11 and Figure 12, SSVR performs better than several traditional methods. In Figure 12d,e, SSVR performs better on the edges of objects with varying color depths, that is, at Positions 1 and 2, and especially at Position 2, SSVR performs significantly better than SVR. The PSNR of SSVMR is 6% higher than that of SVR and 7% for GF. The SNR of SSVMR is 8.9% higher than that of SVR and 11% for GF.

### 3.3. Denoising Color Images

This section extends the application of the algorithm to color images of natural objects in daylight. In color image denoising experiments, different models are trained for each channel and then the individual denoised channel maps are combined into a single image for evaluation. The aim of SSVR is to learn a more precise regression hyperplane by using sample distribution information to capture the relationship between pixels and their neighbors. In the color image, the neighboring pixels of a single channel pixel (*x*,*y*) are distributed not only in its own channel but also in the vicinity of (*x*,*y*) in the other two channels, so the training sample is a vectorization of the pixel cube. The SSVR regression model for each channel uses the same weight distribution.

**Figure 13:**
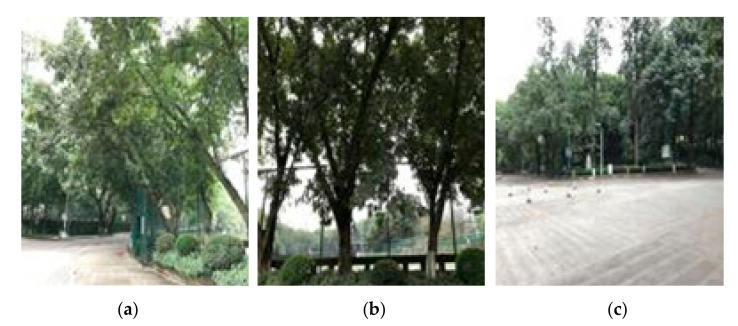
(**a**) Color image C1. (**b**) Color image C2. (**c**) Color image C3.

In the experiment, the pixel values of the RGB channels of each image are used as denoising image features. The size of the window is 3×3, so the training sample extracted from the three channels is a vector of 27×1. The training image is shown in Figure 13a and the test images are shown in Figure 13b,c. The training image contains both dark and light pixels (road surface and trees), while the test image in Figure 13b is dominated by dark pixels and Figure 13c by light pixels.

**Figure 14:**
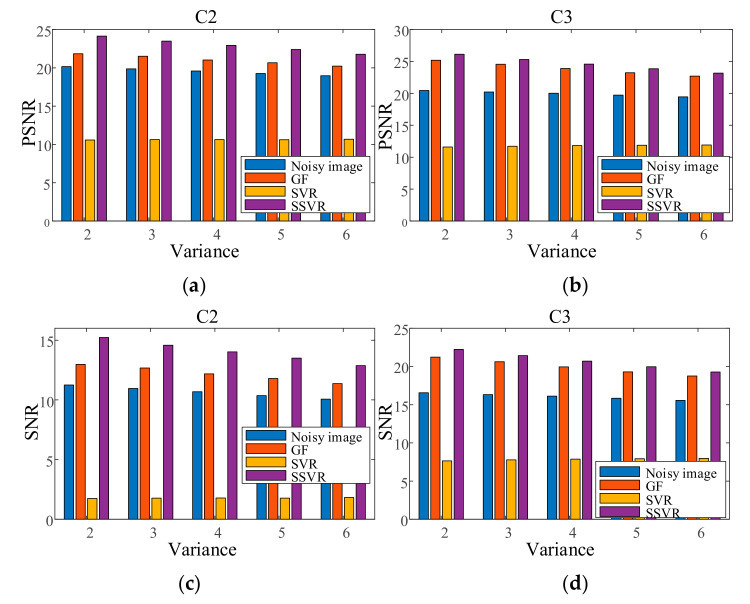
PSNR of different denoising methods with Gaussian noise under different variances on (**a**) C2, (**b**) C3. SNR of different denoising methods with Gaussian noise under different variances on (**c**) C2, (**d**) C3.

**Figure 15:**
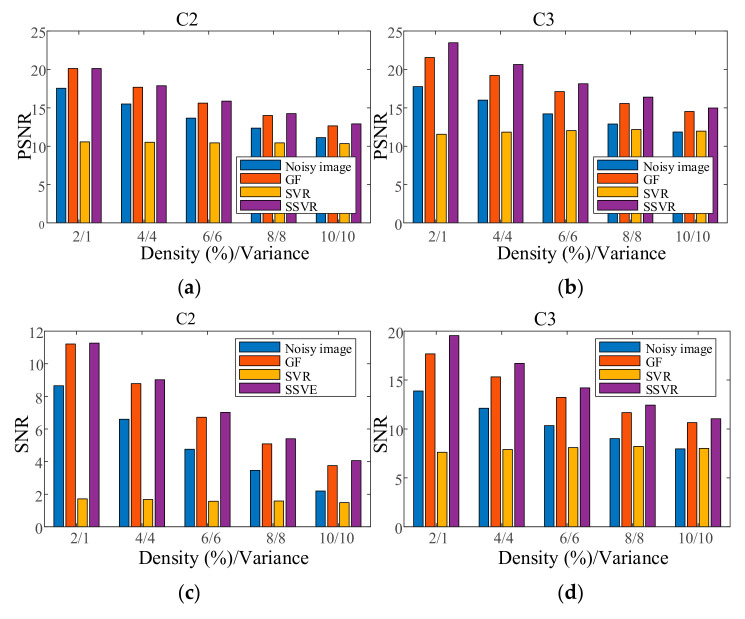
PSNR of different denoising methods with mixed noise under different levels for (**a**) C2, (**b**) C3. SNR of different denoising methods with mixed noise under different levels on (**c**) C2, (**d**) C3.

In the first experiment, each channel of image C1 is contaminated with Gaussian noise with a variance of 0.04 as training data. Each channel of the testing images is contaminated with Gaussian noise variance varying from 0.02 to 0.06. The results are shown in Figure 14. In the second experiment, each channel of the testing images is contaminated with a mixture of Gaussian and salt and pepper noise for each channel, and the results are shown in Figure 15. It can be seen that on color image, the denoising effect of SSVR is obviously better than that of other methods, especially far better than SVR.

**Figure 16:**
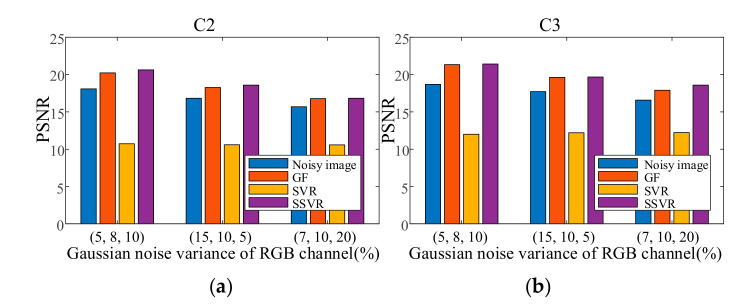

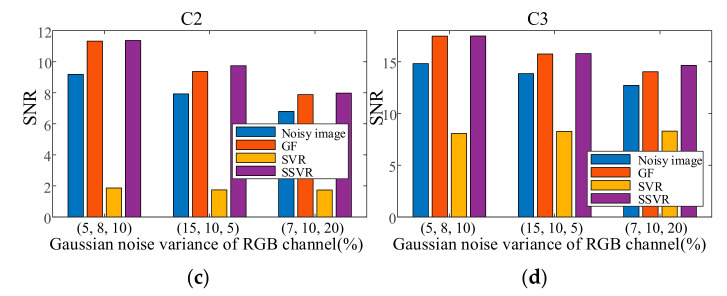
PSNR of different denoising methods with non-uniform noises on (**a**) C2, (**b**) C3. SNR of different denoising methods with non-uniform noises on (**c**) C2, (**b**) C3.

**Figure 17:**
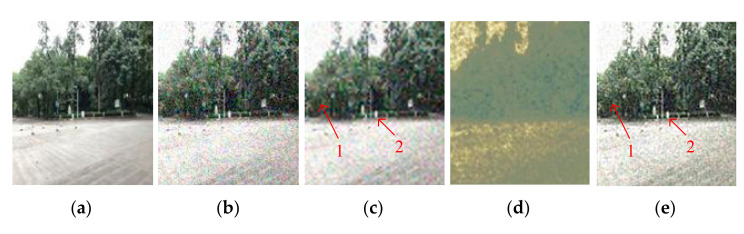
Comparison on C3. (**a**) Original image. (**b**) Noisy image with salt and pepper with density 5%, PNSR = 17.72, SNR = 13.87. (**c**) Denoised image by wavelet, PSNR = 21.22, SNR = 17.37. (**d**) Denoised image by SVR, PSNR = 13.67, SNR = 9.8. (**e**) Denoised image by SSVR, PSNR = 25.87, SNR = 22.

Because non-uniform noise is widespread in multichannel images, we also apply SSVR to non-uniform noise. In the testing images, different channels are added with different degrees of noise. The two results in Figure 16 show that SVR is almost ineffective in denoising in multi-channel color images and even increases pollution. The SVR results are consistent with those shown in Figure 17d. SSVR is slightly more effective than the wavelet method. It can be seen that demising multi-channel images is an issue that deserves further research.

Figure 17 shows the denoising performance obtained by adding 0.5% variance Gaussian noise to each channel of image C3. As can be seen, Figure 17d is completely blurred, which indicates that the SVR is almost ineffective in multi-channel color image denoising. Comparing Figure 17c,e in the Positions 1 and 2, it can be seen that Figure 17e is ahead of Figure 17c in terms of edge definition and color reproduction. The PSNR of SSVMR is 89% higher than that of SVR and 21% for wavelet. The SNR of SSVMR is 124% higher than that of SVR and 26% for wavelet.

## 4. Conclusions

Traditional SVR rarely considers the effect of sample distribution information during the model training process. To overcome this drawback, SSVR improves noise by introducing significance measurement information based on spatial density information as the refining coefficient into SVR. The refining coefficient for the penalty parameter in SVR could improve the importance of normal data and reduce the influence of noisy data in the solution process. As an implementation example of SSVR, a significance factor which utilizes the cutoff distance as a spatial density estimation method was proposed and applied on image denoising. Experimental results demonstrated that the proposed SSVR improved with samples significance information could achieve effective performance on image denoising. Further research will focus on applying the proposed method in wide fields such as bearing vibration signals and acoustic emission signal denoising.

## Data Availability

Not applicable.
